# Concise Review: Methods and Cell Types Used to Generate Down Syndrome Induced Pluripotent Stem Cells

**DOI:** 10.3390/jcm4040696

**Published:** 2015-04-15

**Authors:** Youssef Hibaoui, Anis Feki

**Affiliations:** 1 Department of Genetic Medicine and Development, University of Geneva Medical School and Geneva University Hospitals, 1 Rue Michel-Servet, CH-1211 Geneva, Switzerland; 2 Department of Obstetrics and Gynecology, Cantonal Hospital of Fribourg, Chemin des Pensionnats 2-6, 1708 Fribourg, Switzerland

**Keywords:** induced pluripotent stem cells, Down syndrome, trisomy 21, T21-iPSCs, reprogramming, isogenic iPSCs

## Abstract

Down syndrome (DS, trisomy 21), is the most common viable chromosomal disorder, with an incidence of 1 in 800 live births. Its phenotypic characteristics include intellectual impairment and several other developmental abnormalities, for the majority of which the pathogenetic mechanisms remain unknown. Several models have been used to investigate the mechanisms by which the extra copy of chromosome 21 leads to the DS phenotype. In the last five years, several laboratories have been successful in reprogramming patient cells carrying the trisomy 21 anomaly into induced pluripotent stem cells, *i.e.*, T21-iPSCs. In this review, we summarize the different T21-iPSCs that have been generated with a particular interest in the technical procedures and the somatic cell types used for the reprogramming.

## 1. Introduction

Down syndrome (DS), caused by a trisomy of chromosome 21 (HSA21), is the most common genetic developmental disorder, with an incidence of 1 in 800 live births. DS individuals show cognitive impairment, learning and memory deficits, arrest of neurogenesis and synaptogenesis, and early onset of Alzheimer’s disease [1,2]. They are also at greater risk of developing acute lymphoblastic leukemia (ALL) and acute myeloid leukemia (AML). The incidence of ALL, the most common leukemia in childhood, is approximately 20-fold higher in children with DS than in the general population. The incidence of AML is between 46- to 83-fold higher, with a particular susceptibility to acute megakaryoblastic leukemia [3]. The detailed pathogenetic mechanisms by which the extra copy of HSA21 leads to the DS phenotype remain unknown. However, there is evidence that several regions exist on HSA21 with various “dosage sensitive” genes contributing to a given phenotype, which could also be modified by other genes on HSA21 and in the rest of the genome [4,5].

Several models have been used to recapitulate the DS phenotype, such as mouse models [6]. However, they do not accurately recapitulate the specificities of the human phenotype. A new finding indicating that induced pluripotent stem cells (iPSCs) can be reprogrammed through the introduction of a few factors [7,8] has opened a new avenue for the investigation of neurological diseases (reviewed in [9]). The first application of this technology appeared only one year after the release of these articles, with the derivation of iPSC lines from patients affected by several diseases including trisomy 21 [10]. Since that research paper, a dozen other studies reporting the generation of trisomy 21 iPSCs (T21-iPSCs) have appeared in the last five years. In this concise review, we will summarize the T21-iPSCs that have been reported up to now with a particular focus on the origin of the somatic cells and the procedures used for the reprogramming.

## 2. Procedures Used for the Reprogramming of T21-iPSCs

Direct reprogramming into iPSCs involves the ectopic introduction of a set of core pluripotency-related transcription factors in a somatic cell. In the vast majority of iPSC studies, *OCT4* (also known as *POU5F1*), *SOX2*, *KLF4* and *MYC* (also known as *c-MYC*) are used for the reprogramming into pluripotency as in the original study by Yamanaka’s team [7]. In addition to this so-called OSKM cocktail, Thomson and colleagues also proposed another reprogramming cocktail that comprises *OCT4* and *SOX2* but *NANOG* and *LIN28* instead of *KLF4* and *c-MYC*: the so-called OSNL cocktail [8]. When this process is successful, compacted colonies appeared in the culture dish that showed marked similarities to embryonic stem cells (ESCs) with respect to morphology, growth properties, expression of pluripotency factors, self-renewal and developmental potential [7,8,11]. The current published T21-iPSC lines have been all generated with the OSKM cocktail, except for one study where T21-iPSCs were derived with the OSNL cocktail [12]. Thus, these T21-iPSC lines were derived predominantly through integrative delivery systems and, to a lesser extent, through non-integrative delivery systems (Table 1).

**Table 1:** The different T21-iPSCs reprogrammed.

Type and Age of Donor Cells	Reprogramming Method	Characteristic of the iPSCs	DS Phenotype Investigated	References
Fibroblasts from patients (1 year, 1 month) with unrelated controls	Retrovirus with OSKM	The first T21-iPSCs generated		[10]
Fibroblasts from a DS patient (1 year) with unrelated controls	Retrovirus with OSKM		Neurons and AD associated phenotype	[10,13,14]
Skin fibroblasts from DS patients (childs) with no control	Lentivirus with OSKM	T21-iPSCs with different karyotypes for DS		[15]
Amniotic fluid cells (second trimester) with age match control	Lentivirus with OSKM		Reduced number of neurons	[16]
Fibroblasts from DS individuals	Retrovirus with OSKM	Isogenic iPSCs	Myeloid Leukemia	[10,17]
Neonatal fibroblasts Fetal stromal cells Fetal mononuclear cells	Doxycycline-induced Lentivirus with OSKM, Retrovirus with OSKM		Myeloid Leukemia	[18]
Fibroblasts from DS individuals	Lentivirus with OSNL	Trisomy 21 deletion through TKNEO	Proliferation and neurogenesis	[12]
Fibroblasts from unrelated patients and controls Fibroblasts from a mosaic DS patient.	Episomal vectors with OSK or OSNLM	Non integrating procedures Isogenic iPSCs	Neurogenesis, gliogenic shift	[19]
Fibroblasts from unrelated patients and controls Fibroblasts from a mosaic DS patient	Retrovirus with OSKM Sendai virus with OSKM	Isogenic iPSCs	Neuron deficit	[20]
Fibroblasts from a DS patient (1 year)	Retrovirus with OSKM Sendai virus with OSKM	Trisomy 21 deletion through Xist	Proliferation and neurogenesis	[10,21]
Fetal skin fibroblasts from monozygotic twins discordant for trisomy 21	Lentivirus with OSKM	Monozygotic twins discordant for trisomy 21	Neurogenesis, gliogenic shift, rescue of the phenotype	[22,23,24]
Fibroblasts	Retrovirus with OSKM	Non-isogenic and isogenic iPSCs	Neurogenesis, gliogenic shift	[25]

DS: Down syndrome; iPSCs: induced pluripotent stem cells; reprogramming cocktails: O for OCT4, S for *SOX2*, K for *KLF4*, M for *c-MYC*, N for *NANOG*, L for *LIN28*.

### 2.1. Integrative Procedures Used for the Derivation of T21-iPSCs

The first T21-iPSC lines were generated with the OSKM cocktail using the Maloney murine leukemia virus (MMLV)-derived retroviruses pMXs [10]. MMLV-derived retroviruses have been used in more than half of the studies reporting the generation of T21-iPSCs (Table 1). In this respect, MMLV-derived retroviruses allow the delivery of genes into the genomes of dividing cells, and the efficiency of iPSC generation from human fibroblasts using MMLV-derived retroviruses is approximately 0.01%.

Lentiviral vectors have also been successfully used to reprogram T21-iPSCs (Table 1). They are generally derived from HIV. They exhibit higher infection efficiency than MMLV-derived retroviruses and allow the delivery of genes into the genome of dividing and non-dividing cells. The efficiency of iPSC generation from human fibroblasts using lentiviral vectors is comparable to those of MMLV-derived retroviruses (~0.01%). However, compared to MMLV-derived retroviruses, lentiviruses are less repressed in human pluripotent stem cells (hPSCs) [26]. In this respect, a major improvement has been seen in the method with the development of single polycistronic vectors containing all the reprogramming factors, which reduce multiple transgene insertion into the genome [27]. Moreover, in one study, T21-iPSCs were derived through doxycycline-induced lentiviral vectors with an OSKM cocktail [18]. The main advantage of this method is that it allows greater control over transgene expression; compared with constitutive lentivirus, in which the vector is integrated and then may or may not be silenced, the doxycycline-induced lentivirus is integrated and silenced when doxycycline is removed. A more recent improvement of the method has been the introduction of lentiviral vectors that incorporate loxP sites allowing their excision via Cre recombinase when pluripotency is achieved [28]. However, viral elements flanking the loxP sites still remain after excision.

The use of integrating vectors offers a more efficient means of reprogramming but also raises major drawbacks with the risk of (i) genetic and epigenetic aberrations; (ii) overexpression of potentially tumorigenic genes such as *c-MYC*; and (iii) incomplete silencing of reprogramming factors following differentiation. Also, the use of integrative approaches has been associated with genomic instability of the generated iPSCs. Genomic instability in iPSCs could come from various sources, which means karyotype analysis is one of the first verifications that has to be done when establishing an iPSC-based disease model. Mutations can originate from the parental somatic cells from which the iPSCs are derived or can be generated during the reprogramming process [29]. However, this is still debated, as growing evidence supports a similar frequency of genetic aberrations in iPSCs, independently of the reprogramming method (integrative or non-integrative) or the cell type used for the reprogramming [29,30,31,32,33]. Alternatively, it could be acquired after culture adaptation and passaging over time [34,35]. For example, mechanical passaging appears to produce more stable cells with a normal karyotype than enzymatic harvesting methods [36,37,38]. This genomic instability is not restricted to long-term culture, but can appear very rapidly, within five passages after switching human ESCs to enzymatic dissociation [39].

Another major concern of integrative delivery systems is related to a possible transgene reactivation that could lead to the overexpression of potentially tumorigenic genes such as *c-MYC* or *KLF4*. For instance, the presence of *c-MYC* is a major limitation, as chimeras derived from iPSCs frequently develop tumours due to the reactivation of *c-MYC* [40,41]. Therefore, transgene silencing has to be investigated after initial expansion of a few passages of the newly generated iPSCs. Moreover, early reports have proposed that residual transgene expression (of *c-MYC* or *KLF4* in particular), after using integrating viral approaches may affect pluripotency and differentiation states [8,11]. It is important to note, however, that reprogramming approaches that exclude *c-MYC* are more labor-intensive and less efficient. In fact, *c-MYC* is an important inducer of reprogramming [42,43,44,45], activating pluripotent genes and maintaining the pluripotent state of PSCs [46,47,48]. It is considered the driver of the first transcriptional wave during cellular reprogramming into iPSCs [49]. This could explain, at least in part, why the vast majority of the reported iPSC lines are achieved using *c-MYC*. Of note, other potential contributors of tumorigenicity of iPSCs have been reported; in particular, we highlighted the crucial role of *NANOG* during reprogramming into iPSCs with respect to germ cell tumor formation [50].

Regarding the impact of these methods on the differentiation potential of iPSC lines, Hu *et al.* reported variable potency of iPSCs to differentiate into neural cells independently of the set of reprogramming transgenes used to derive iPSCs as well as the presence or absence of the reprogramming transgenes in the generated iPSCs [51]. In line with this, in a study comparing the differentiation potential of iPSC lines derived from a single parental fibroblast line via several reprogramming strategies (+/− *c-MYC*, excised or non-excised transgene), neither the presence of *c-MYC* nor the presence of the transgene removed the *in vitro* potential of these iPSCs to differentiate into neuroprogenitor cells, neurons, astrocytes and oligodendrocytes [52]. Furthermore, it appears that omission in iPSCs of reprogramming factors, and of *c-MYC* in particular, compromises the efficiency of their subsequent differentiation into neuroprogenitor cells and neurons [53].

### 2.2. Non-Integrative Procedures Used for the Derivation of T21-iPSCs

Two non-integrative approaches have been used for the generation of T21-iPSCs: episomal vectors [19] and Sendai virus vectors [20]. Briggs *et al.* reported the first generation of T21-iPSCs free of vectors and transgenes [19]. This reprogramming was achieved by transfection with oriP/Epstein-Barr nuclear antigen-1 (oriP/EBNA1)-based episomal vectors [54]. These plasmids can be transfected without the need for viral delivery and can be removed from cells by culturing in the absence of selection. In other terms, the exogenous DNA is not integrated into the iPSC genome. However, the reprogramming efficiency of this approach for human fibroblasts is extremely low, ~0.0006% [54].

An alternative non-integrative method has been used for the generation of T21-iPSCs by the mean of Sendai virus [20]. Sendai virus, a member of the Paramyxovirus family is an enveloped virus with a nonsegmented negative-strand RNA genome. Modified Sendai virus (through the deletion in one of the two envelope glycoproteins) has emerged as an efficient and robust RNA-based gene delivery system. Since Sendai virus RNA replication occurs in cytoplasm of the infected cells without a DNA phase, there is no risk of vector genome integration into host genome [55]. Thus, the efficiency reached by this method is much higher than that achieved with episomal vectors for the reprogramming of human fibroblasts to iPSCs: ~1% [55].

## 3. Age and Type of the Donor Cells Used for the Reprogramming

Reprogramming into iPSCs requires the delivery of pluripotency factors into a somatic cell. This is achieved with different efficiencies and kinetics depending on the donor cell type. Therefore, the choice of the type of the donor cells is an important aspect to consider before the generation of disease-specific iPSCs. As for 80% of the studies reporting the derivation of human iPSCs, fibroblasts remain the cell type the most commonly used for the derivation of T21-iPSCs (Table 1). There are many reasons for this. Even though dermal fibroblasts are obtained from skin biopsies or neonatal foreskin biopsies, which require invasive procedures, they present several advantages. First, the culture of fibroblasts is relatively easy and cheap. In culture, fibroblasts also exhibit a high proliferation rate, viability and stability (at least in low passages, as the risk of accumulated genomic alteration increases with passaging). Moreover, the discovery of iPSC technology has been done initially in mouse fibroblasts [56] and subsequently adapted in human fibroblasts [7,8]. Then, most of the data available on the relative kinetics and efficiencies of the different methods used for the reprogramming have been characterized using fibroblasts as donor’s cells (reviewed in [57]). In line with this, most of the iPSCs banked have been generated with fibroblasts as a starting material. All these considerations make fibroblasts as the main cell type used for the reprogramming in general as well as in DS research. However, other cell type has been used for the generation of T21-iPSCs such as cells from amniotic fluids which are more easily obtained and reprogrammed into iPSCs [16]. Indeed, second semester amniocenteses are routinely collected in the context of prenatal diagnosis screening. Also, compared with fibroblasts, cells from amniotic fluids transduced with OSKM exhibited higher efficiency (100 times more) and are reprogrammed into pluripotency more than twofold faster [58]. This makes cells from amniotic fluids as easy to reprogram as keratinocytes [59]. Similarly, fetal stromal cells and mononuclear cells have been used for the generation of T21-iPSCs [18].

During the reprogramming process, the epigenetic state of the donor’s cells has to be reset to obtain a pluripotent state; this includes modification of the DNA methylation profile, and chromatine marks [60,61]. However, genome wide DNA methylation studies showed that iPSCs retain the DNA methylation signature of the donor’s cells [60,62]. This so-called “epigenetic memory” consists of residual specific marks of the parental somatic cells that escape the reprogramming process, leading to a preferential differentiation potential of the generated iPSCs into the tissue of origin rather than other lineages [60,61]. For instance, iPSCs derived from cord blood display a higher capacity for hematopoietic differentiation than iPSCs derived from keratinocyte, and reciprocally [60]. However, it is important to note that studies investigating donor epigenetic memory of iPSCs have confounded the donor’s cell type and the donor genetic background due to the practical difficulty of collecting various primary tissues from the same donor. Also, it has been reported that donor epigenetic memory appears to be gradually lost after prolonged iPSC culture [60,62,63], which supports the idea that the preferential differentiation potential due to epigenetic memory can be overcome. Moreover, there are some indications that non-coding RNAs such as miRNAs play a role in maintaining residual memory of donor cells in iPSC-derived cells [64,65]. For instance, miR-155 have been identified as a key player in somatic donor memory of iPSCs in the context of iPSC differentiation toward hematopoietic progenitors [64].

Another important factor that should be considered when deriving disease specific iPSCs is the age of the donor’s cells. T21-iPSCs have been generated from DS tissue from fetal, neonatal and adult stages (Table 1). In this respect, embryonic tissue appears to be more prone to reprogramming into pluripotency than adult tissue. Barriers such as the age and the differentiation status of the donor’s cells could explain this property [66,67,68]. For instance, it has been shown that the increased levels of the age-related genes *p16* (*INK4A*), *p19* (*ARF*) and *p15* (*INK4B*), which encodes two tumor suppressors, limit the efficiency and the fidelity of the reprogramming [67]. Also, the differentiation stage of the starting cell used for the reprogramming has a critical impact on the efficiency of reprogramming into iPSCs. Blood progenitors reprogram into iPSCs up to 300 times more efficiently than terminally differentiated blood cells [68]. Similarly, neural progenitor cells which express *SOX2* endogenously have only been successfully reprogrammed into iPSCs with *OCT4* [69]. Considering that donor cell type and age may affect the differentiation potential of the iPSCs, it is crucial to establish D21-iPSCs and T21-iPSCs from the same parental somatic cells at the same developmental age.

## 4. Isogenic D21-iPSCs and T21-iPSCs

Among the potential variables that must be considered when establishing an hPSC-based disease model, the definition of a non-disease control is of crucial importance [70,71]. The genetic background of both control and the affected cells has to be identical or similar in order to be sure that the differences observed in the studies are due only to the disease and not to the choice of either the control or the affected samples. Traditionally, iPSCs from unrelated healthy individuals together with ones from age-matched, unrelated affected patients are often used to decrease the variability of individual genetic background and the variability among the iPSC lines regarding their *in vitro* differentiation potential. To overcome these problems, several approaches have been developed to obtain isogenic D21-iPSCs and T21-iPSCs. This is particularly important as isogenic D21-iPSCs and T21-iPSCs represent an ideal situation for the investigation of the effect of the supernumerary HSA21 on the DS phenotype, since the rest of the genome is theoretically identical. It could also limit the need to generate several iPSC lines.

**Figure 1:**
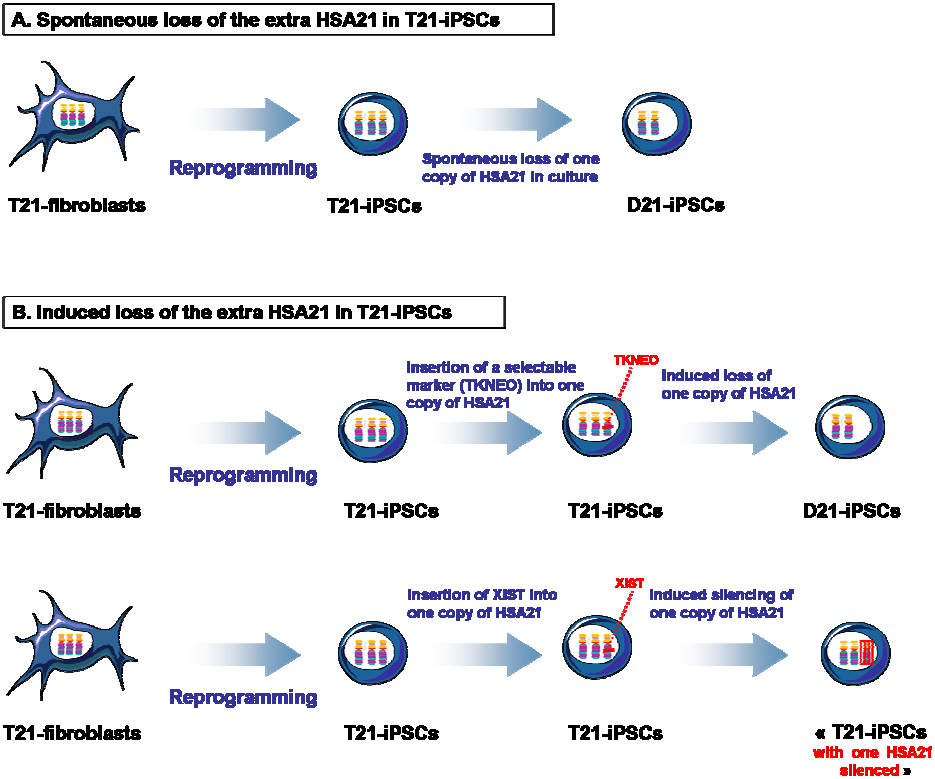
Isogenic iPSCs obtained through spontaneous or induced loss of trisomy 21. Isogenic D21-iPSCs and T21-iPSCs have been obtained either via spontaneous or induced loss of one copy of HSA21. (**A**) T21-iPSCs can lose one copy of HSA21 after culture adaptation and passaging over time [17,25]; (**B**) The loss of one copy of HSA21 in T21-iPSCs has been induced through the insertion of a foreign gene called *TKNEO* into one copy of HSA21 (within the *APP* gene) of T21-iPSCs. When these T21-iPSCs were grown in a medium that selected against *TKNEO*, the most common reason for the cells to survive was the loss of one copy of HSA21 [12]. The silencing of one copy of HSA21 in T21-iPSCs has been induced through the insertion of *XIST* into one copy of HSA21 of T21-iPSCs. This leads ultimately to the generation of isogenic D21-iPSCs [21].

Chromosomal aberrations have been often observed after culture adaptation over time in hPSCs [34]. In particular, stable genomic aberrations that confer growth, self-renewal, and differentiation advantages for hPSCs are often selected over time [29,34,72]. In the study by MacLean *et al*., one clone of T21-iPSCs lost one copy of HSA21 with culture passages leading to a mixed culture of isogenic D21-iPSCs and T21-iPSCs. Then, they succeeded in isolating isogenic D21-iPSCs and T21-iPSCs from this mixed culture by cultivating them as single cells and discriminating D21-iPSCs from T21-iPSCs by FISH analysis (Figure 1A) [17]. This event seemed to occur also for one clone of T21-iPSCs generated by Chen *et al*. [25].

In another study, Li *et al.* succeeded in deriving isogenic D21-iPSCs from T21-iPSCs. For this, they used an adeno-associated virus to introduce a *TKNEO* transgene into one copy of HSA21 of T21-iPSCs. When the T21-iPSCs were grown in a medium that selected against *TKNEO*, the only cells that survived were the ones that spontaneously lost the extra HSA21 (Figure 1B) [12].

In an elegant study, Lawrence *et al.* have shown that the extra copy of HSA21 in T21-iPSCs can be silenced through the insertion of the RNA gene called *XIST*, a gene responsible for the silencing of one of the two X-chromosomes in female cells. Interestingly, they demonstrated that the insertion of *XIST* gene at a specified location in the HSA21 using zinc finger nuclease technology effectively repressed genes across the supernumerary HSA21 in T21-iPSCs, leading to the generation of isogenic D21-iPSCs and T21-iPSCs (Figure 1B) [21].

**Figure 2:**
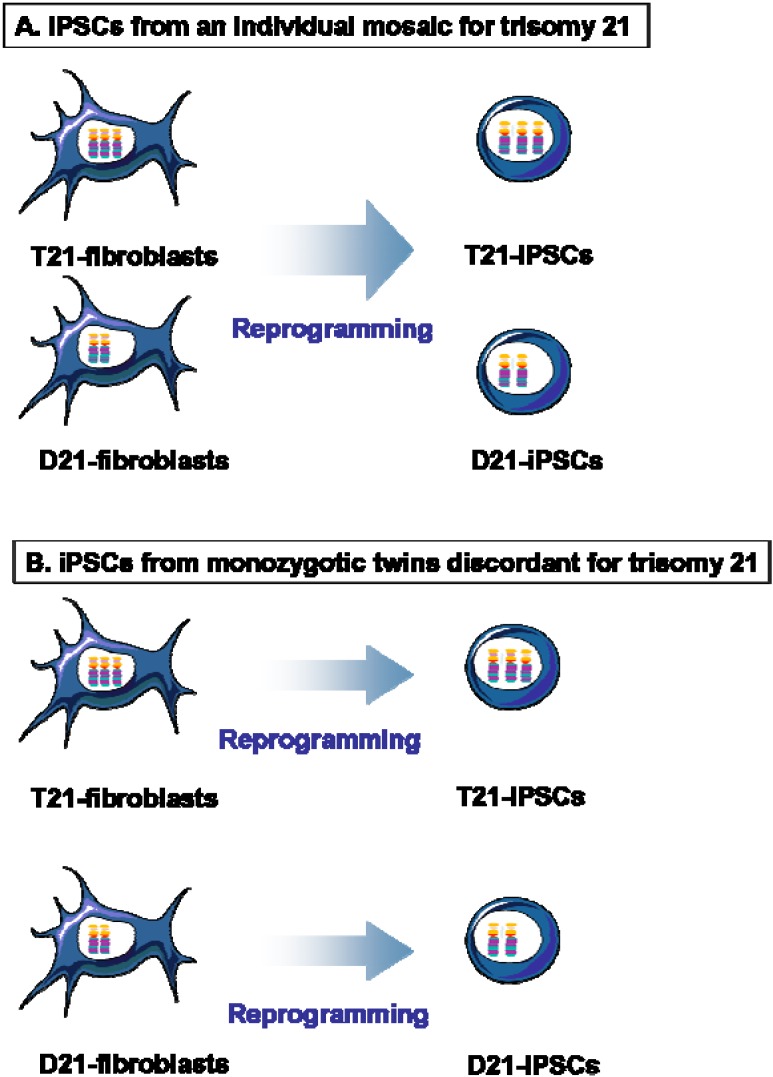
Isogenic iPSCs from individual mosaic for trisomy 21 or from monozygotic twins discordant for trisomy 21. (**A**) Isogenic D21-iPSCs and T21-iPSCs have been derived from mosaic patients for trisomy 21 [19,20]; (**B**) Isogenic D21-iPSCs and T21-iPSCs have been generated from monozygotic twins discordant for trisomy 21 [22,23,24].

It is well known that varying degrees of mosaicism for trisomy 21 may exist in the generation population; it represents 1%–3% of DS cases [73]. This leads to a combination of euploid cells and cells carrying trisomy 21 anomaly within individual tissues (reviewed in [74]). Taking advantage of this rare situation, two recent studies reported the derivation of isogenic D21-iPSCs and T21-iPSCs from fibroblasts from an individual mosaic for trisomy 21 (Figure 2A) [19,20].

Most monozygotic twins are “genetically identical” and are in general expected to be concordant for health, chromosomal abnormalities, and Mendelian disorders. However, in very rare cases, monozygotic twins can be discordant for the disease (reviewed in [75]). One example of this is monozygotic twins discordant for trisomy 21 [76]. We exploited this rare and unique situation by deriving iPSCs from fetal fibroblasts of monozygotic twins discordant for trisomy 21 [22,23,24] and thus confounding effects from genomic variability were theoretically eliminated (Figure 2B).

## 5. Down Syndrome Phenotype Investigated

Among the phenotypes observed in DS individuals, only two have been explored using T21-iPSCs, namely brain-related defects and myeloid leukemia.

### 5.1. Brain-Related Defects

Five groups, including our own, have reported the recapitulation of the relevant DS phenotype using neurons derived from T21-iPSCs. Consistent with a DS post-mortem human brain, T21-iPSCs showed reduced neurogenesis when induced to differentiate into neuroprogenitor cells (NPCs) and further mature into neurons [19,21,23]. This effect was associated with a proliferation deficit and increased apoptosis of NPCs derived from T21-iPSCs [23]. Thus, together with the reduced neurogenesis, T21-iPSCs showed a greater propensity to generate both astroglial [19,23] and oligodendroglial cells [23] upon neural induction and differentiation. This gliogenic shift appeared early in development as it starts at the NPC level [23]. Moreover, neurons derived from T21-iPSCs exhibited not only a reduction of their population but also structural alterations compared to those derived from D21-iPSCs. They exhibited in particular reduced dendritic development [23] and reduced expression of synaptic proteins such as synapsin or SNAP25 [20,23]. In line with this, we found a lower proportion of excitatory glutamatergic synapses whereas the proportion of inhibitory GABA-ergic synapses was not substantially altered in neurons derived from T21-iPSCs [23]. Regarding the electrophysiological properties, neurons derived from T21-iPSCs displayed a significant synaptic deficit that affects excitatory glutamatergic synapses and inhibitory GABA-ergic synapses equally [20].

Furthermore, the increased proportion of astroglial cells at the expense of neurons upon neural induction and differentiation of T21-iPSCs [19,23] is of special interest as it has been shown that astrocytes derived from T21-iPSCs exhibited higher levels of reactive oxygen species (ROS) and lower levels of synaptogenic molecules than astrocytes derived from D21-iPSCs. This ultimately contributes to oxidative stress-mediated cell death and abnormal maturation of neurons derived from T21-iPSCs [25].

Finally, Shi *et al.* used T21-iPSCs as a PSC model of Alzheimer’s disease pathology, given that DS individuals present early onset of Alzheimer’s disease. They showed that cortical neurons derived from T21-iPSCs exhibited greater secretion of amyloid peptides, tau protein phosphorylation and cell death, supporting the notion that T21-iPSCs are an excellent model for AD study [13].

### 5.2. Myeloid Leukemia

Two recent studies have explored the potential of T21-iPSCs to model hematopoietic defects associated with trisomy 21 [17,18]. Using a differentiation protocol that mainly drives hPSCs towards primitive yolk sac-type hematopoietic progenitors, Chou *et al.* showed that hematopoietic progenitors derived from T21-iPSCs exhibit an increased propensity for erythropoiesis [18], similar to what it is observed in DS fetal liver hematopoiesis [77,78]. However, in contrast with DS fetal liver hematopoiesis, no difference was found between D21-iPSCs and T21-iPSCs in their capacity to generate megakaryocytes [18]. In the second study, MacLean and colleagues used a differentiation protocol that drives hPSCs towards definitive fetal-liver type progenitors. They found that hematopoietic progenitors derived from T21-iPSCs (and from T21-ESCs) exhibit higher multi-lineage colony-forming potential [17]. In particular, T21-iPSC-derived hematopoietic progenitors showed a greater colony-forming unit for erythroid, myeloid and megakaryocyte lineages [17], consistent with DS fetal liver hematopoiesis [77,78]. This indicates that trisomy 21 favours the expansion of hematopoietic progenitor cells. Altogether, these two studies point to different defects in primitive yolk sac-type hematopoietic progenitors and definitive fetal-liver type progenitors derived from T21-iPSCs and further suggest that the effects of trisomy 21 are likely specific to the developmental stages of the hematopoietic progenitors. Further studies using this iPSC-based model should provide important clues regarding the impact of trisomy 21 on hematopoietic development.

## 6. Conclusions and Perspectives

Since the first paper demonstrating that fibroblasts from DS patients can be reprogrammed into iPSCs by retroviral delivery of OSKM cocktail [10], several alternative methods and cell types have been used to generate T21-iPSCs (Table 1). At the moment, there is no consensus for the cell type that should be used for the reprogramming. The choice of the starting material depends not only on the availability of the cell type, but also on the ability and efficiency of these cells for reprogramming. With respect to the reprogramming method that should be used, this depends mostly on the priorities regarding the applications of the generated iPSCs. The priorities are not the same if the generated iPSCs aimed at investigating (i) the reprogramming mechanisms; (ii) disease modelling and drug screening and (iii) regenerative medicine. For the former aim, as the reprogramming approach needs to be efficient, the integrative inducible lentiviruses will meet most of the requirements. The safety of the generated iPSCs is a major requirement for clinical applications but less crucial for disease modelling and drug screening studies. In this respect, Sendai viruses and mRNA methods offer the advantage of generating iPSCs free of vectors and transgenes with a high efficiency [79].

Another major concern when generating iPSCs is the definition of a non-diseased control. In most of the studies reporting disease modelling using iPSCs, iPSC lines from unrelated healthy donors have been used as controls since genetically matched non-diseased controls are often difficult to obtain. In this respect, isogenic D21-iPSCs and T21-iPSCs offer the unique opportunity to study the effect of the supernumerary HSA21 on DS phenotype without the biological “noise” that could result from the variability of individual genetic background. These isogenic D21-iPSCs and T21-iPSCs has been achieved via several ways: (i) by spontaneous or induced loss of one copy of HSA21 in T21-iPSCs [12,17,21,25]; (ii) isogenic D21-iPSCs and T21-iPSCs from an individual mosaic for trisomy 21 [19,20]; (iii) isogenic D21-iPSCs and T21-iPSCs from monozygotic twins discordant for trisomy 21 [22,23,24]. Of note is the recent progress in genomic editing technologies such as transcription Activator-Like Effector Nucleases (TALEN), Zinc Finger Nucleases (ZFN) and Clustered Regularly Interspaced Short Palindromic Repeats (CRISPRs) (for review [80]) should provide opportunities to investigate genotype-phenotype correlations using “gene-edited” iPSC lines. For instance, it should allow the study of the contribution of candidate genes on DS phenotype by the investigation of the effect of genetic loss-of-function in T21-iPSCs and gain-of-function in D21-iPSCs of HSA21 genes in the target cell type of interest for DS.

A major drawback of iPSC technology is the variability that can appear at each step of the reprogramming and the differentiation processes. Reprogramming into iPSCs can give rise to unpredictable alterations of the genome such as copy number variants, karyotypic abnormalities, point mutations and deletions, epigenetic memory of the parental somatic cells [29,30,31,32,33,34,35,36,37,38,39,60,61,62,63]. Therefore, it is possible that such genetic and epigenetic alterations can affect the fidelity of the results regarding disease modeling and drug screening. Also, there is evidence that iPSC lines display variable potency to differentiate into the cell type of interest [51,60]. However, it is unclear what factors contribute to this variable efficiency of the iPSC differentiation, as it appears independent of the methods used for the reprogramming [51]. For this reasons, it is important to generate several iPSC lines from accurately chosen tissue of multiple normal and DS individuals, using them in priority non-integrative procedures. Such efforts will improve the identification of the pathogenetic mechanisms involved in DS by reducing the noise that could result from the variability of individual genetic background and from the experimental artifacts. At the same time, it will reduce the discovery of false pathogenetic mechanisms.

Another aspect that should be taken into account in DS modelling using iPSCs is the presence of a broad phenotypic variability among DS individuals. Even though DS individuals share some morphogenetic characteristics [1,4,5], trisomy 21 can have differential pathogenicity on individual genomes [81]. For example, brain-related defects are common traits in all DS individuals but other traits such as congenital heart defects only occur in ~40% of them. In line with this, cases of partial trisomy 21 and other HSA21 rearrangements associated with DS features have been reported [4,5]. Such cases could serve to link genomic regions of HSA21 with specific phenotypes given the possibility of generating the target cell type of interest for DS using T21-iPSCs.

Regarding the applications of T21-iPSCs, the abundance of studies reporting the generation of T21-iPSCs clearly shows that T21-iPSCs are reliable tool for DS modelling, given that the protocols for differentiation of iPSCs into neurons or hematopoietic cells are available. These protocols enable the production of large quantities of the target cell type for DS modelling. Some of these studies have been successful in recapitulating DS phenotypes using iPSCs (see Table 1). In this respect, transcriptional profiling of T21-iPSCs has proven extremely informative for the study of the pathogenetic mechanisms involved in DS phenotype [17,18,19,22,23,24]. For example, T21-iPSCs recapitulate the developmental disease transcriptional signature of DS [22,23,24]. Furthermore, T21-iPSCs allow the possibility of linking the genetic data to biological insights by deciphering the molecular changes in the target cell type of interest for DS (reviewed [82]). Then, the causal involvement of candidate HSA21 genes and pathways can be assayed by studies involving genetic loss-of-function in T21-iPSCs and gain-of-function in D21-iPSCs through genomic editing methods (for review [80]). Regarding DS modelling, only two phenotypes have been investigated so far: brain-related defects and myeloid leukemia (Table 1). However, other phenotypes associated with DS deserve investigations (heart defects, lymphoid leukemia and others). Moreover, modelling DS using iPSCs offers opportunities for drug screening. In concert with functional genomics, iPSCs form a powerful cellular model platform for drug screening assays with direct relevance to the DS phenotype. Integrating the genetic findings and the functional insights obtained from T21-iPSC-derived cells should provide a path to predict which drug might best counteract DS phenotype. Four studies have produced the proof of concept of such an application. Several proteins or pathways have been targeted and demonstrated beneficial effects on the DS phenotype, including oxidative stress-mediated cell death (with *N*-acetylcysteine, an antioxidant) [19], neurogenesis impairment (with epigallocatechine gallate, a DYRK1A inhibitor) [23], the gliogenic shift (with monocycline, an anti-inflammatory drug) [25] and AD-related phenotype (with inhibitors of gamma secretase) [13]. Finally, one promising aspect of iPSC technology is the potential use of these cells in cell replacement therapy to treat neurological diseases [9]. However, iPSCs have not been used until recently for clinical applications due to concerns over the immunogenicity and tumorigenicity of these cells [83,84]. Recently, iPSC technology has generated enthusiasm in the field of cell replacement therapy with the decision of Takahashi’s team to treat a patient with a degenerative eye disease [85]. The possibility to induce the loss of one copy of HSA21 in T21-iPSCs and to produce subsequently isogenic D21-cells offers great hope for the treatment of some DS phenotypes (such as brain-related defects). However, numerous challenges remain for cell replacement therapy [9,86], and further studies are needed to address to which extent cells derived from iPSCs can be used for DS therapy. The coming years will tell whether these cells fulfil their potential.

In conclusion, we believe that T21-iPSC-derived cells are an invaluable resource for medical research. They will advance our understanding of the pathogenetic mechanism by which the extra copy of HSA21 leads to the DS phenotype. They have already offered the first opportunity to study the developmental events in the cell type of interest for DS: brain-related defects using iPSC-derived neurons and leukemia using iPSC-derived hematopoietic cells. IPSCs could also serve as a cellular platform for the evaluation of potential therapeutics.
